# Supplementary material for: k-mer manifold approximation and projection for visualizing DNA sequences
Source: Genome Res. 2025 May;35(5):1234–46. doi: 10.1101/gr.279458.124 (PMC12047656; doi:10.1101/gr.279458.124)
Supplement: Supplement 8 [file Supplemental_Data_1.zip › Supplemental_Data_1/POU1F1_TACACC40NCAC_AI_4/POU1F1_TACACC40NCAC_AI_4_MDS.pdf]

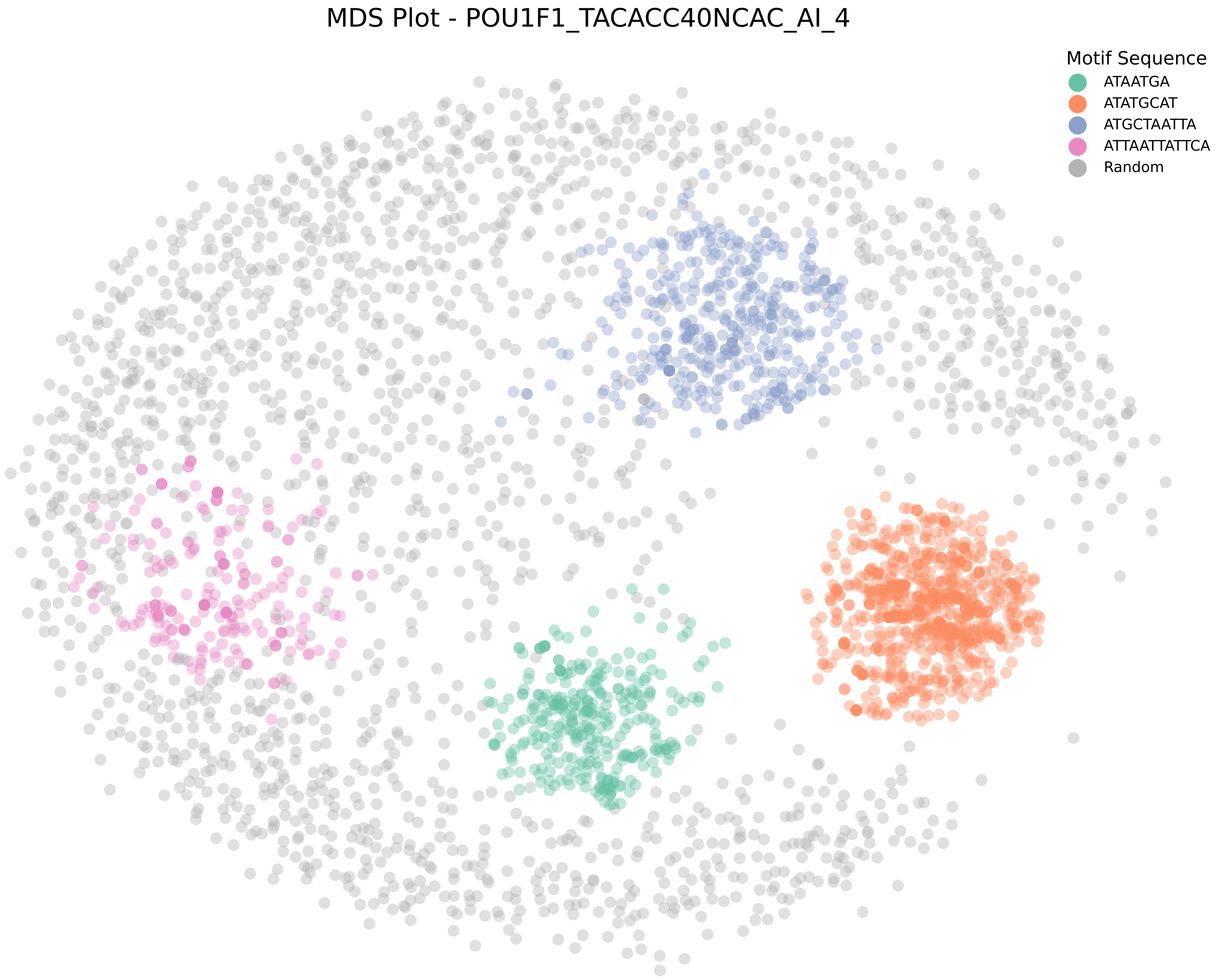
MDS Plot - POU1F1_TACACC40NCAC_AI_4
Motif Sequence
ATAATGA
ATATGCAT
ATGCTAATTA
ATTAATTATTCA
Random
